# Finding abundance regulators

**DOI:** 10.7554/eLife.83907

**Published:** 2022-11-03

**Authors:** Olivia Ozguc, Sibylle Vonesch

**Affiliations:** 1 Center for Microbiology, VIB-KU Leuven Leuven Belgium; 2 Centre of Microbial and Plant Genetics, Department of Microbial and Molecular Systems, KU Leuven Leuven Belgium

**Keywords:** protein regulation, crispr screen, base editor, gene regulatory network, gapdh isoenzymes, ras/pka, *S. cerevisiae*

## Abstract

A new pooled screening method in yeast allows scientists to probe how protein levels are regulated by mutating thousands of genes at once.

**Related research article** Schubert OT, Bloom JS, Sadhu MJ, Kruglyak L. 2022. Genome-wide base editor screen identifies regulators of protein abundance in yeast. *eLife* **11**:e79525. doi: 10.7554/eLife.79525.

Each cell in our body contains over 10,000 different proteins that perform the activities the cell needs to live. But how do cells know how much of each protein to make? The information needed to make each protein is encoded in our genes, which are used as templates to make messenger RNAs (mRNAs) in a process called transcription. The mRNAs are in turn translated into proteins by ribosomes. Each of these steps is executed and tightly controlled by several proteins, which together form a regulatory network that determines the amounts of a protein that get synthesized. For most proteins, we only know certain parts of this network, making it difficult to predict the factors that affect protein levels in the cell, which can play a role in disease.

Geneticists can identify which proteins help to regulate protein levels in the cell by inactivating individual genes to see whether stopping the production of the protein coded for by that gene affects the levels of another protein. Unfortunately, doing this for each regulator protein individually is expensive and time-consuming. So-called 'pooled screens' can survey many inactivating mutations at once in a single tube full of cells, but it is challenging to read out the effects of these perturbations on protein levels. Advances in single-cell RNA sequencing have allowed scientists to link specific mutations to changes in mRNA levels (Datlinger et al., 2017). However, mRNA levels are not always good predictors for protein abundance, because several cellular mechanisms that regulate protein abundance act only after transcription (Vogel and Marcotte, 2012). Some of these mechanisms can even buffer the effects of genetic mutations, such that a change in mRNA level due to a mutation does not lead to a change in protein level (Buccitelli and Selbach, 2020).

Now, in eLife, Olga Schubert, Leonid Kruglyak and colleagues at the University of California, Los Angeles report on a new pooled screening approach to directly study how protein abundance is regulated in the yeast *Saccharomyces cerevisiae* (Schubert et al., 2022). To generate the necessary inactivating mutations in the yeast cells, the team used a DNA base editor – a molecular tool that can be directed to convert one type of DNA base to another at specific sites determined by a targeting sequence (a so-called gRNA). The editor was originally developed for human cells (Komor et al., 2016), so Schubert et al. first had to show that it could efficiently make these mutations in yeast. They did this by using the editor to inactivate genes necessary for survival in yeast, which led to the cells dying.

Next, Schubert et al. developed an approach to read out the effect of inactivating mutations on the levels of 11 proteins of interest (Figure 1). First, they generated 11 strains of yeast each expressing a protein of interest fused to a fluorescent protein. These strains allowed Schubert et al. to have a straightforward readout of protein abundance: for proteins engineered this way, fluorescence can be used as a proxy for protein abundance. Next, the base editor was used to introduce inactivating mutations for all yeast genes in these strains, with each cell only receiving mutations at one target site encoded by the gRNA. After editing, cells with high or low fluorescence were isolated using a method called fluorescence-activated cell sorting, and the identity of the perturbed genes determined by sequencing of the gRNA.

**Figure 1.**
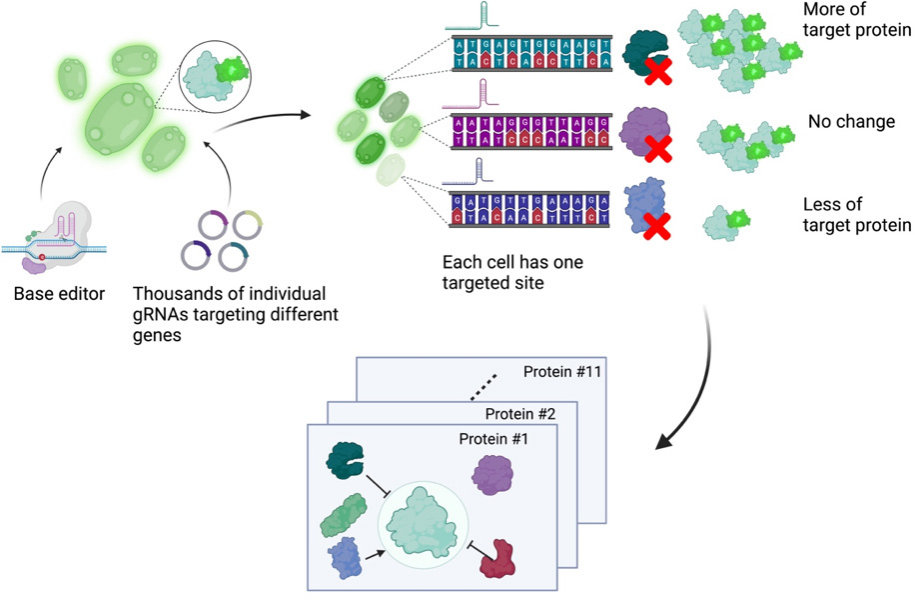
A base editor screen to investigate regulatory relationships affecting protein levels. Left: a library of thousands of guide RNA plasmids (gRNA, circles with sections in different colours) targeting genes across the genome are introduced, along with the base editor, into an engineered yeast strain containing the genetic code for a protein of interest fused to the fluorescent protein GFP. Centre: each cell has a different target gene disrupted by the base editor (shown as DNA sequences in different colours), resulting in a specific protein (the green, purple and blue shapes next to the sequences) not being produced in each cell. Right: after editing, each cell will have different levels of fluorescence depending on which gene was disrupted in it. The top cell has higher fluorescence than an unedited cell because the disrupted gene in it codes for a protein that normally lowers the levels of the protein of interest. The centre cell has the same fluorescence levels as an unedited cell because the disrupted gene in it codes for a protein that does not regulate the levels of the protein of interest. The bottom cell has low levels of fluorescence because the disrupted gene in it codes for a protein that normally increases the levels of the protein of interest. The cells are then sorted depending on their fluorescence levels, and the identity of the gene mutated in each cell is determined by sequencing the gRNA. Bottom: this pooled screening approach allows scientists to define regulatory networks that determine the abundance of each target protein and reveals general properties of networks governing protein expression.

Figure created using BioRender.com

The results showed that mutations that only affected one or two proteins of interest were often found in genes coding for transcriptional regulators. On the other hand, mutating genes that make key components for the translation machinery reduced abundance of most of the proteins under study. This implies that genes involved in translation are more likely to be hubs in protein regulatory networks. Schubert et al. also found that mutations that affected more proteins of interest were also more likely to reduce protein abundance and decrease cellular fitness, since they were often found in genes needed for survival.

Besides confirming known regulatory relationships, Schubert et al. also found many new ones. For example, they propose a new regulatory pathway that links the cell’s ability to sense extracellular amino acids to the activation of the stress-responsive protein Yhb1, increasing stress resistance. The experiments also identified distinct regulatory proteins for different forms of GAPDH, a multifunctional enzyme known for its role in glucose metabolism. This suggests that each form of the enzyme may be required in different metabolic states. GAPDH is an important human enzyme, and it can interact with proteins implicated in neurodegenerative diseases, so understanding how it works and how it is regulated has broad implications beyond yeast (Butterfield et al., 2010).

The findings of Schubert et al. highlight how identifying new regulators, even for well-studied proteins, is important for basic science. In the future, coupling this method with tools that introduce mutations that do not fully inactivate the target genes (Roy et al., 2018; Sharon et al., 2018) may allow a more nuanced view of how protein abundance is encoded in the genome. This, combined with improvements in the speed and sensitivity of protein quantification (Messner et al., 2021; Brunner et al., 2022), may soon allow scientists to map regulators for thousands of proteins at once. Integrating Schubert et al.’s approach with methods like high-throughput imaging or phospho-proteomics (Humphrey et al., 2018) will also increase our understanding of proteins that are mainly regulated through phosphorylation or by their localization in the cell. Overall, the ability to assess more proteins in diverse environments and organisms will deepen our understanding of the regulatory principles that govern protein expression.
